# Supplementary material for: Hofmeister Series for Conducting Polymers: The Road to Better Electrochemical Activity?
Source: Polymers (Basel). 2023 May 26;15(11):2468. doi: 10.3390/polym15112468 (PMC10255087; doi:10.3390/polym15112468)
Supplement: Supplementary file 1 [file polymers-15-02468-s001.zip › polymers-2400341-supplementary.pdf]

Supporting Information

Hofmeister Series for Conducting Polymers: The Road to Better Electrochemical Activity?

Rostislav V. Apraksin[1,*], Alexey I. Volkov[2],

1 - Ioffe Institute, 26 Politekhnicheskaya str., St. Petersburg, 194021, Russia

2 - Department of Electrochemistry, Institute of Chemistry, St. Petersburg State University, 7/9 Universitetskaya Embankment, St. Petersburg 199034, Russia; grulfex@gmail.com

*e-mail: apraksinrv@ioffe.mail.ru

Supporting Information

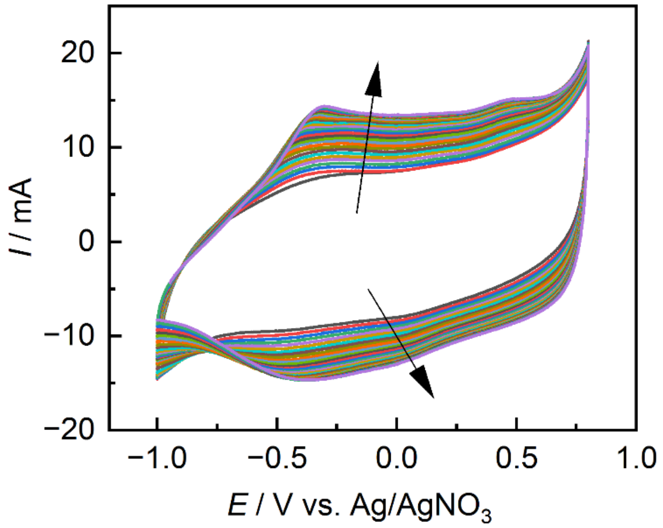

Figure S1. CV of PEDOT:PSS/H_2O film at 50 mV s^{-1} in Et_4NBF_4/AN, 30 cycles

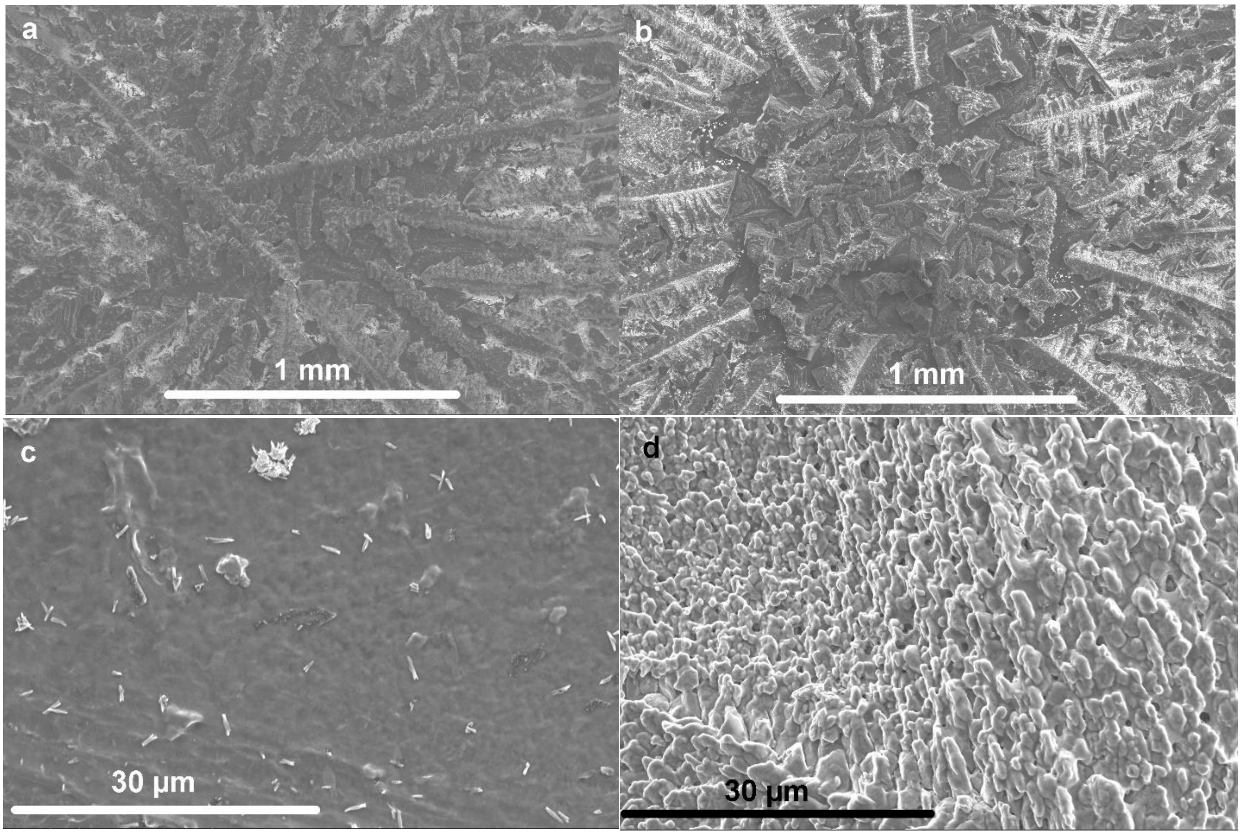

Figure S2. SEM images of a) PEDOT:PSS/NaCl, b) PEDOT:PSS/KCl, c) PEDOT:PSS/$NaClO_4$, d) PEDOT:PSS/$NaNO_3$

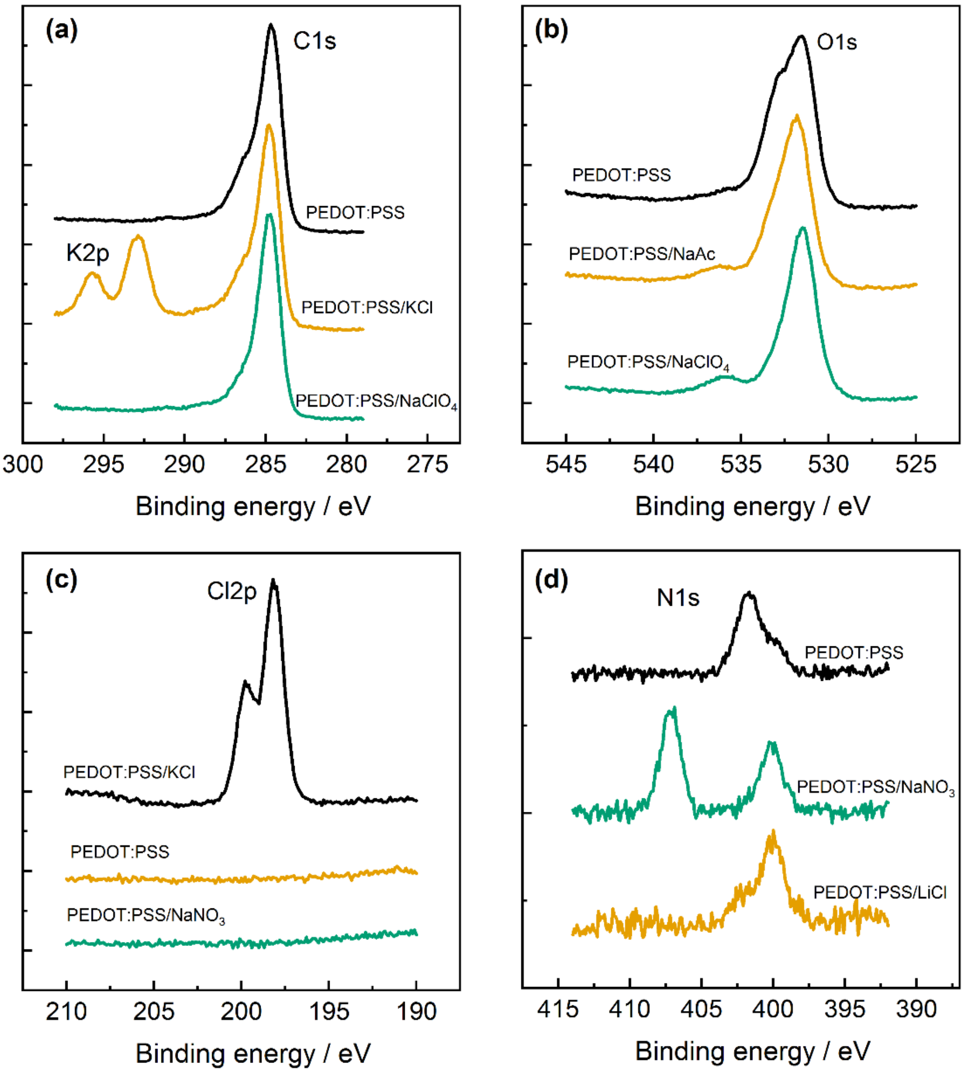

Figure S3. XPS spectra for PEDOT:PSS films: a) C1s core level spectra, b) O1s core level spectra, c) Cl2p core level spectra, d) N1s core level spectra

Supporting Information

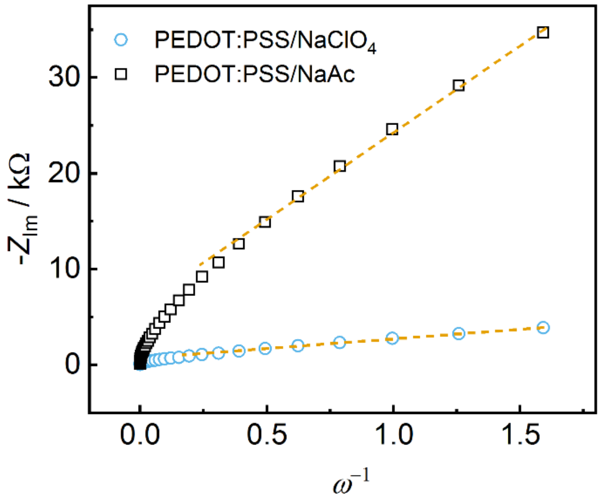

Figure S4. $-Z_{Im} - \omega^{-1}$ dependence at −0.5 V for PEDOT:PSS/NaClO$_4$ and PEDOT:PSS/NaAc

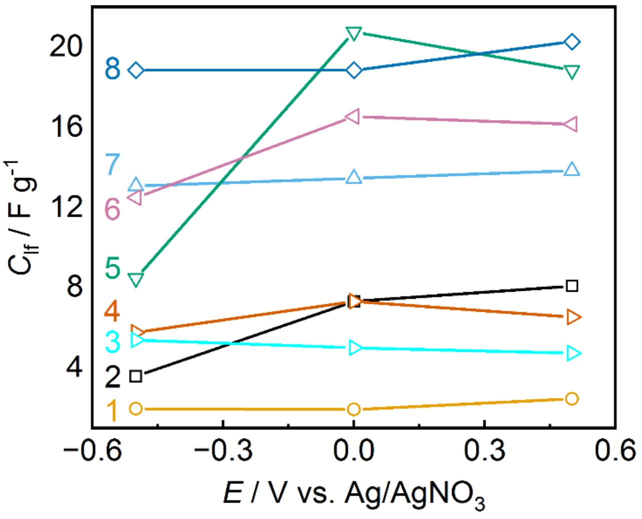

Figure S5. Capacitance calculated from EIS data for 1) PEDOT:PSS/NaAc, 2) PEDOT:PSS/H_2O, 3) PEDOT:PSS/KCl, 4) PEDOT:PSS/NaCl, 5) PEDOT:PSS/LiCl, 6) PEDOT:PSS/$MgCl_2$, 7) PEDOT:PSS/$NaNO_3$, 8) PEDOT:PSS/$NaClO_4$

Supporting Information

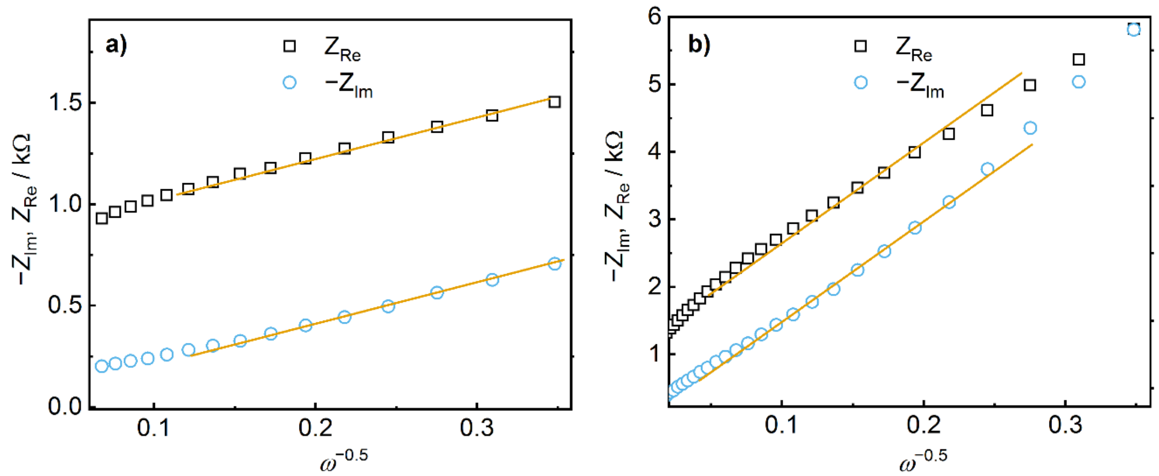

Fig S6. $-Z_{Im}$, Z_{Re} – $\omega^{-0.5}$ dependence at −0.5 V for a) PEDOT:PSS/$NaClO_4$, b) PEDOT:PSS/NaAc

Table S1. Electrochemical parameters obtained from CV measurements.

Additive	OCP, V	E_a, V	E_c, V	C, F g^{-1}
$LiClO_4$	0.4	−0.2	−0.5	37
LiCl	−0.1	−0.2	−0.4	33
$LiBF_4$	0.3	−0.2	−0.5	34
Li_2SO_4	0.1	−0.2	−0.8	28
$NaClO_4$	0.1	−0.2	−0.4	28
NaCl	0.0	−0.1	−0.2	9
NaSCN	0.1	−0.2	−0.4	25
$NaNO_3$	0.4	—	—	13
NaF	0.0	—	—	7
NaAc	−0.2	—	—	7
$MgCl_2$	0.1	−0.1	−0.7	29
$Mg(ClO_4)_2$	0.2	−0.2	−0.4	30
$MgSO_4$	0.1	−0.3	−0.8	28
KCl	0.0	—	—	7
